# Second language acquisition effects of a primary physical education intervention: A pilot study with young refugees

**DOI:** 10.1371/journal.pone.0203664

**Published:** 2018-09-17

**Authors:** Mirko Krüger

**Affiliations:** Division of Social Sciences in Sports, University of Duisburg-Essen, Essen, North-Rhine Westfalia, Germany; Central European University, HUNGARY

## Abstract

**Background:**

A physical education (PE) intervention for young refugees was designed combining physical activity within the context of primary PE games with second language learning activities in German. The intervention was based on theoretical implications from the field of second language acquisition and evidence for positive effects of physical activity on cognitive outcomes (e.g. language acquisition). The aim of this study was to analyze short term effects on second language acquisition.

**Methods:**

Sixty-one young refugees were included in the study (*age*: 8.5 ± 1.4 years). The intervention group participated in language-enriched PE lessons based on an elaborated approach to second language learning acquisition. The control group did not receive any treatment. Both groups were pre-and post-tested in domain specific vocabulary, listening comprehension and use of local prepositions within the context of primary PE games.

**Results:**

Results from linear mixed-effect modelling suggest that the intervention group significantly improved domain specific vocabulary and listening comprehension in comparison to the control group.

**Conclusions:**

The intervention was successful since the PE lessons contributed to the second language acquisition of young refugees. Therefore, this learning approach might also be useful for physical activity based second language learning activities in other PE contexts for early second language learners in primary school.

## Introduction

The number of refugees who, due to conflicts or persecution, left their home country fleeing to other countries is at the highest level in recorded history [1]. Many of them are children who are still required to attend school [2]. This increase of refugees also affects Germany and the whole German school system [3]. Since 2015 many German schools face the challenge to integrate these new students in everyday school routines to meet their academic needs as well as their wellbeing needs. Among others (e.g. enhancing students´ general knowledge), teachers are asked to foster refugee children’s academic performance, especially their German language proficiency (L2). Therefore, specific L2 learning activities are implemented into regular school routines. That is, individual language courses implemented additional to regular classes and integrated language learning [4], implemented within regular classes, even within Physical Education (PE) classes.

To date, knowledge on the integration of L2 learning is scarce in German literature on PE [5] and only few methodological approaches for PE and L2 language integrated learning (intPE-L2) exist [5,6]. All of them consider the promotion of basic interpersonal communication skills (BICS) and cognitive academic language proficiency (CALP) [7] as overall learning goals incorporating several learning strategies from the field of L2 learning literature [8]. However, these intPE-L2 approaches have not yet been examined regarding their effectiveness within school-based interventions. Of note, intervention studies from early childhood education showed significant improvements of children’s oral and written language acquisition after integrating PE and L2 language learning activities [9,10]. Similar effects have also been found for preschoolers with speech impediment [11]. Furthermore, there is a plethora of evidence indicating that increased physical activity (PA) leads to better academic achievement [12–15], which is also evident for different language learning skills [16–17].

The theory of brain-based learning is suitable to explain these effects. The theory states that moderate to vigorous physical activity (MVPA) effectively stimulates the brain [18]. In short-term, PA leads to immediate biochemical changes in cerebral regions thereby increasing attention and enhancing cognitive performance [19]. In long-term, regular MVPA fosters angiogenesis, neurogenesis, and synaptogenesis in brain areas that are important for the process of learning and memory [19–20]. Additionally, researchers verified both positive short and long-term changes in PA intervention programs for children [21–24]. These findings suggest that regular or additional PE lessons might stimulate children’s cognitive capacities (e.g. L2 learning of young refugees) within iPE-L2 approaches. Despite this evidence, no study has yet examined such effects among refugee children at primary school age.

We hypothesized that intPE-L2 learning activities in additional PE lessons may influence domain specific vocabulary learning, listening comprehension and use of local prepositions within the context of primary PE classes. In a quasi-experimental study, we therefore investigated the effectiveness of a school-based intPE-L2 learning approach for young refugees. We predicted improved domain specific vocabulary learning, listening comprehension and use of local prepositions after the intervention. The intervention was supposed to result in a better performance than mere schooling and maturation (control group). This study is the first of this kind in Germany and should therefore be regarded as an exploratory analysis.

## Methods

### Participants

Sixty-one young refugees took part in the study (34 males and 27 females, mean age 8.45 ± 1.37 years). The refugees were chosen from different primary schools in the city of Essen (North Rhine-Westphalia, Germany). Inclusion criterion for the study participation was immigration to Germany in consequence of fleeing conflict or persecution [25]. For reasons of practicability the participants could not be randomly assigned to the study conditions (intervention vs. control group). The intervention group (18 males and 13 females, aged 6–10 years), consisted of young refugees from six different countries (Spain, Iraq, Italia, Romania, Syria and Uganda). Students from this group immigrated to Germany between 2015 and 2016. 16 male and 14 female refugees (aged 6–11 years) from eleven different countries (Afghanistan, Azerbaijan, Spain, Greece, Italia, Nigeria, Syria, Romania, Bulgaria, Bosnia-Herzegovina, Croatia) served as controls. Students from this group immigrated to Germany between 2013 and 2016. The study was conducted in accordance to the declaration of Helsinki [26] and approved by the ethics committee of the Medical Faculty of the University of Duisburg-Essen (No. 17-7738-BO). All parents and students provided their written informed consent before taking part in the study.

### Intervention

#### Procedure

Trained teacher apprentices supervised the interventions. All of them obtained a degree in a pedagogic discipline (Bachelor of Education). Before the intervention started they received extensive training from the author and other staff members regarding the theoretical concepts and intervention contents. The intervention took place from August to December during the first school term in 2016/2017 and was announced as an additional physical education program for young refugees. Participants did not know about the study background. The intervention consisted of six lessons (duration: 90 minutes each). Language-enriched PE activities were included in all parts of the lessons, i.e., during warm up, main part, and during review and closure. Table 1 displays the specific contents during the intervention lessons. To ensure accuracy and preciseness of the intervention, a primary teacher was present during the intervention sessions and apprentices were asked to prepare a self-report after the lessons. Each individual intervention session was conducted by at least two of the apprentices. The control group did not receive any intervention and both, the intervention and the control group, were tested pre and post of the intervention period.

**Table 1:** Content of the intervention lessons “Exploring popular primary PE games”.

PE lesson contents (L2 focus)
1. Stop Game (Listening comprehension: e.g. *Spin in a cycle*)
2. Chicken ball (Domain specific vocabulary: e.g. *team*, *line*, *throw*)
3. “Fisher, fisher! How deep is the water?” (Listening comprehension: e.g. *Hop on one leg*)
4. “Every man for himself” (Domain specific vocabulary: e.g. *wall bars*)
5. Climbing island (Domain specific vocabulary: e.g. *gymnastic mats*)
6. „Searching for the right card” relay (Domain specific vocabulary: e.g. *gymnastic ball*; and prepositions, e.g. *upon*)
7. „We want to move”relay (Domain specific vocabulary: e.g. *gymnastic ball*; and prepositions: e.g. *upon*)
8. Burning ball (Domain specific vocabulary for game rules and used equipment)
9. Varying Burning ball (Use of local prepositions: e.g. *behind*)

#### Intervention approach

The intervention was based on an integrated Physical Education and L2-learning approach that combines PE integrated L2 learning strategies from the field of sport pedagogy [5,6] as well as major principles of usage-based approaches to second language acquisition [8].

Second language acquisition (SLA) is based on extensive L2 input. The more learners are exposed to the target language the more and the faster they will learn to use it [27]. Research from the field of SLA shows that L2 learners agree on the importance of *frequency and richness of input* for developing the highly implicit knowledge that is needed to communicate effectively in the L2 [8,28]. This way, L2 learners implicitly internalize the rules of the target language. Often used linguistic elements (e.g. vocabulary or abstract frames on sentence level) are learned faster than rarely used elements. These linguistic elements are pattern ranging from simple morphems like *-ing* to complex and abstract frames on sentence level like the imperative. Linguistic elements form a structured inventory of a speaker´s knowledge in the convention of their L2 (a semantic network).

As indicated by L2 research findings, SLA is an *explicit and implicit learning process* [29]. Explicit learning describes scenarios in which L2 learners are instructed to actively look for specific patterns (i.e., intentional learning). Implicit learning means that L2 students derive knowledge from complex, rule-governed stimuli without intending to or being aware of it. The intervention used in the present study was conceptualized as being an implicit and explicit learning scenario. Nevertheless, L2 learning can happen in diverse learning contexts in and outside school. Therefore, we selected linguistic elements that were specific enough to the PE content domain to allow the examination of intervention effects. Thus, we decided to focus on constructs on word level (i.e., domain specific vocabulary and use of local prepositions) and sentence level (i.e., listening comprehension) that are often used within the intervention context and that would have a low probability to be found outside the intervention context. The additional PE lessons were used as authentic and relevant opportunities to communicate. Furthermore, these communication opportunities were embedded and enriched with physical activities for stimulating L2 learning [12–17]. Against this background, we designed intervention lessons to support the use of local prepositions, providing diverse occasions for communicating, support movements and verbal activity (i.e., combining speaking and moving). The main goal of the intervention was to foster second language acquisition with the help of physical activity within additional L2-learning enriched PE lessons.

### Outcome measures

**Fig 1:**
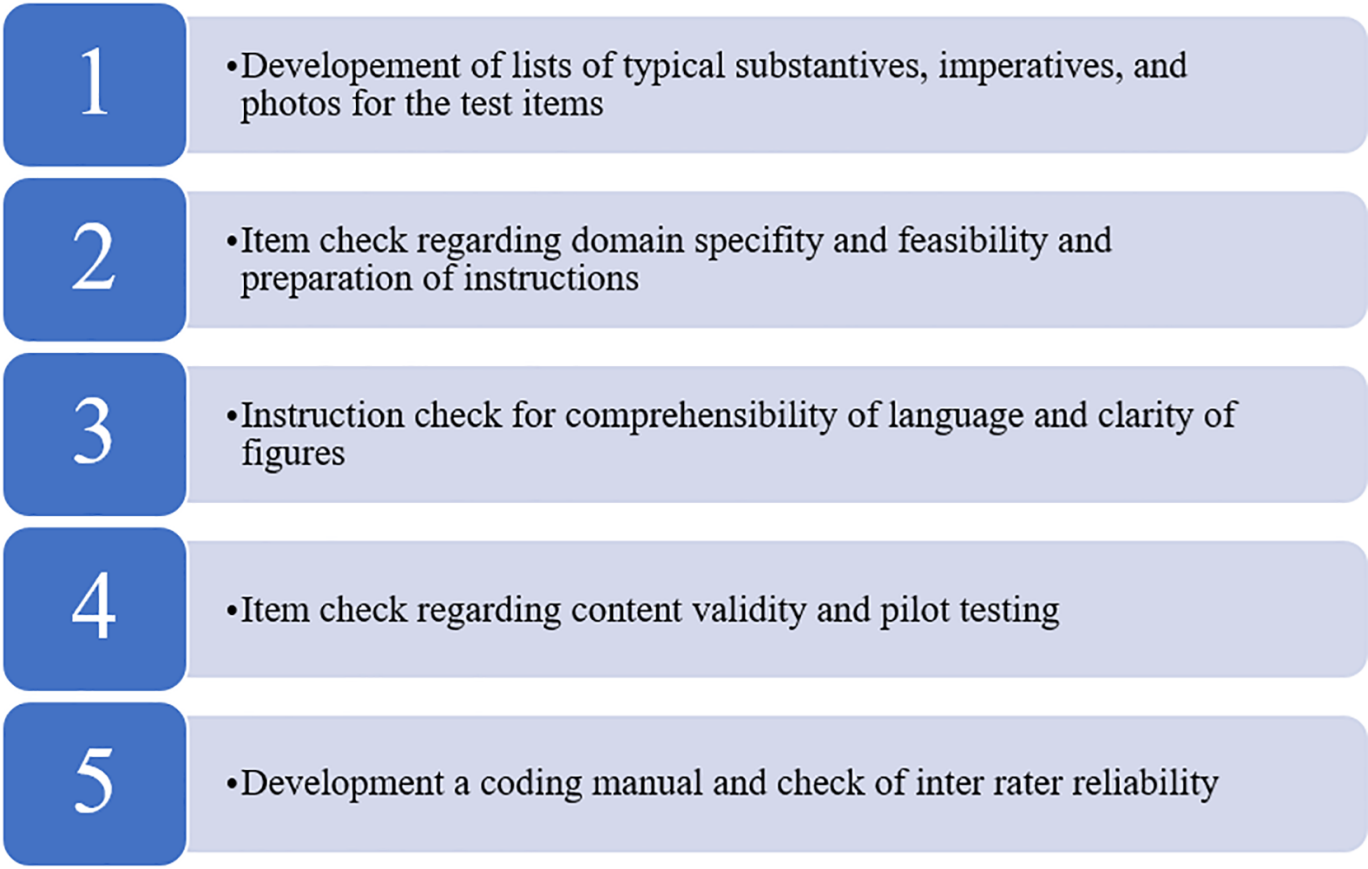
Flow chart representing the language test development steps.

To test students’ domain specific vocabulary learning, listening comprehension and use of prepositions we developed three test batteries using a multiple steps approach for language test development and examining strategies [30]. The whole process is represented in Fig 1.

Firstly, a list of typical substantives (→ domain specific vocabulary; e.g. “Weichbodenmatte” [*engl*.: soft flor mat]) and a list of typical imperatives (→ listening comprehension; e.g. “Dreh dich im Kreis” [*engl*.: spin in a cycle]) were developed. Also, we took many photos of a young girl standing in different positions next to a piece of gymnastics apparatus (e.g. a bench; → use of prepositions). To meet the strategy of rich input, the lists and figures contained linguistic elements (e.g. substantives), that would frequently occur within the context of primary PE games and would often be used during the intervention. Secondly, the lists and figures were checked by five PE experts (doctoral students, postdocs and professors) regarding their domain specificity and feasibility. At the end of this step, all linguistic elements were transferred into three standardized test batteries along with suitable verbal instructions. The test batteries for domain specific vocabulary and for use of prepositions were carried out by verbal and figural instructions. Thirdly, the instructions of all test batteries were checked for comprehensibility of language and clarity of figures. At the end of this step, ten domain specific vocabulary items, five listening comprehension items, and five prepositions items remained. In the next step, the final instrument was content validated by an expert from the field of second language acquisition research at the University of Duisburg-Essen and pilot tested. For this purpose, the instrument was utilized in three young refugees who had to show good usage of the instrument within the testing situation. Lastly, the author of the present study constructed a coding manual with rules for correct (= 1 point) and incorrect (= 0 point) answers for all outcome dimensions. The manual was developed based on the principles of standardized test items batteries and with the help of the audio recorded pilot tests to identify good examples and precedents for correct and incorrect answers. With the help of this manual 20% of the tests were double coded by two teacher trainees. Inter rater reliability for the coding was Cohen´s *κ* = .89 showing an almost perfect agreement [31]. The means of the outcome dimensions in pre-and post-tests were used to assess second language acquisition within the field of primary PE games.

### Data analysis and control variables

All data were screened for missing data and outliers: across both sub-samples (intervention and control group), none of the variables had missing values.

The data analysis consisted of two parts: a preliminary analysis and a main analysis to discover the intervention effects. Descriptive statistical analysis was used to analyze the characteristics of the sample. A chi-square test for gender and a multivariate analysis of variance (MANOVA) for the other control variables were conducted with the collected pre-test data. This was done to examine statistical significant differences in the outcomes and control variables between the two groups prior to the intervention. Gender, age, basic motor competencies [32] and cognitive ability (the German version of SON-R 6–40; [33]) were chosen as control variables because they represent potentially important moderators in learning contexts (e.g. [34–37]). Although there are several cognitive ability tests that could be administered to a large group of people, we decided to use the SON-R 6–40 with an individual testing procedure because it is a fair assessment tool for children from cultural minorities [38]. It can be conducted without using written or spoken words, and it consists of four subtests (analogies, mosaics, categories and patterns). It is a valid and reliable intelligence test for children and adults from the age six to forty [33]. Further data (age, gender, immigration year to Germany) were recorded using a self-prepared questionnaire filled out by the children with the help of the teacher trainees, translators and the author of the study during audio-recorded face-to-face interview. Proficiency in the German language was judged by the class teachers at A1-Level (beginners) of the Common European Framework of Reference for Languages for all children within the intervention and control group. After immigrating to Germany, they all attended so called integration classes with intensive German language teaching (15-20h per week) in addition to their normal school routine.

The main analysis was performed to observe the intervention effects using linear mixed effect-models (LMM). We decided to use this class of statistical models to deal with dependency of a subject´s residuals that challenges researchers when using data from repeated measures and longitudinal studies. LMM use likelihood-based techniques to estimate means, variances, regression coefficients, and standard errors including random subject effects to account for the influence of subjects on their repeated observations. LMM can deal with balanced and unbalanced designs as well as autocorrelation by allowing to specify different base-lines and slopes for each of the children with regard of the dependent variables (further [39–40]).

## Results

### Preliminary analysis

The MANOVA results reflected no statistically significant differences at multivariate level (*F*(3, 57) = 1.34, *p* = .26; *η*^2^ = .07) for any of the investigated dependent variables. For the independent variables (see Table 2) significant differences were observed (*F*(1, 59) = 2.59, *p* = .04; *η*^2^ = .18). Subsequent ANOVAs yielded statistically significant differences regarding age (*F*(1, 59) = 8.96, *p* = .00; *η*^2^ = .13): the intervention group was younger (*M* = 7.96; *SD* = 1.16) than the control group (*M* = 8.96; *SD* = 1.40). Therefore, age was considered in the following analyses. No statistical significant differences were identified for cognitive ability (*F*(1, 59) = 0.68, *p* = .41; *η*^2^ = .01). Both groups presented similar ability values (intervention: *M* = 84.43; *SD* = 12.44; control: *M* = 81.20; *SD* = 15.11). Both basic motor competencies locomotion (*F*(1, 59) = 1.46, *p* = .23; *η*^2^ = .02) and object control (*F*(1, 59) = .24, *p* = .61; *η*^2^ = .00) did not show significant differences. Males and females were also equally represented in the intervention and control group (*χ*^*2*^(1) = .01; *p*< .05).

**Table 2:** Descriptive statistics (%, mean, SD) on independent variables separated by treatment groups.

Variable		N	Intervention Group	N	Control Group
Immigration to Germany		30	-	31	-
	Year 2013	-	-	1	3.22
	Year 2014	-	-	6	19.35
	Year 2015	17	56	15	48.38
	Year 2016	13	44	9	29.03
Common home language = non-German		30	100	31	100
Gender = female		30	43.33	31	45.16
Age		30	7.96 (1.16)	31	8.96 (1.40)
Cognitive ability		30	83.70 (10.79)	31	81.30 (14.86)
Basic motor competencies		30	-	31	-
	Locomotion	-	3.48 (2.03)	-	4.22 (2.15)
	Object control	-	3.67 (1.68)	-	3.73 (1.83)

Bold type: p < 0.05; SD = standard deviation.

### Effects of the intPE-L2 intervention

Descriptive analyses revealed differing baseline values between the conditions at pretests and differing error variances between and within conditions. Descriptive statistics of the outcome measures are displayed in Table 3. We performed a linear mixed effect model using R, Version 3.4.0 [41], and the function lmerTest from the packages lmerTest [42] to analyze the effectiveness of the intPE-L2 intervention. As fixed effects time (t1 = pretest, t2 = posttest], age and group (1 = IG, 2 = CG) as main effects and two interaction effects (group x time and age x group) were entered into the LMM. As random effects, intercepts and slopes as subjects for the effect of measurement were used. Tables 4–6 displays results of those dependent variables that are central for testing our hypothesis. The significance level was set at *p*≤ .05.

**Table 3:** Descriptive statistics on outcome measures separated by treatment groups and times of testing (T1 = pretest, T2 = posttest).

Condition		T1		T2	
		*M (SD)*	Median	*M (SD)*	Median
Domain specific vobabulary					
	Intervention Group	2.59 (1.86)	2.00	5.07 (1.92)	5.00
	Control Group	3.26 (2.18)	3.00	4.14 (2.30)	4.00
Listening comprehension					
	Intervention Group	3.11 (1.60)	4.00	4.22 (1.36)	5.00
	Control Group	3.19 (1.89)	4.00	3.38 (1.79)	4.00
Use of prepositions					
	Intervention Group	1.11 (1.45)	1.00	1.88 (1.71)	1.00
	Control Group	1.84 (1.64)	2.00	2.15 (1.86)	2.00

**Table 4:** Result of the linear mixed model for domain specific vocabulary.

						*95% Confidence Interval*	
Parameter (fixed effects)	*β*	SE(*β*)	*df*	*t*	p	Lower	Upper
Intercept	5.15	0.80	52.10	6.43	.000	3.54	6.76
Time [= t1]	-0.95	0.28	59.00	-3.33	.001	-1.52	-0.38
Age [= 6]	-3.17	1.93	50.00	-1.63	.108	-7.07	0.71
Age [= 7]	-0.36	1.18	50.00	-0.30	.758	-2.75	2.01
Age [= 8]	-1.05	1.07	50.00	-0.98	.329	-3.21	1.09
Age [= 9]	-0.88	1.00	50.00	-0.88	.382	-2.91	1.13
Age [= 10]	-2.07	1.07	50.00	-1.93	.059	-4.22	0.08
Group [= 1]	0.11	1.92	50.80	0.05	.953	-3.74	3.97
Time [= t1] x Group [= 1]	-1.49	0.40	59.00	-3.72	.000	-2.29	-0.69
Age [= 6] x group [= 1]	0.76	2.72	50.00	0.28	.779	-4.70	6.24
Age [= 7] x group [= 1]	0.40	2.25	50.00	0.18	.857	-4.11	4.93
Age [= 8] x group [= 1]	1.25	2.13	50.00	0.58	.560	-3.04	5.54
Age [= 9] x group [= 1]	0.88	2.07	50.00	0.42	.672	-3.28	5.05

Random effect: intercepts and slopes for the time effect.

**Table 5:** Result of the linear mixed model for listening comprehension.

						*95% Confidence Interval*	
Parameter (fixed effects)	*β*	SE(*β*)	*df*	*T*	p	Lower	Upper
Intercept	3.80	0.66	50.00	5.73	.000	2.47	5.13
Time [= t1]	-0.15	0.13	59.00	-1.13	.260	-0.43	0.12
Age [= 6]	1.20	1.62	50.00	0.74	.463	-2.05	4.45
Age [= 7]	-0.30	0.99	50.00	-0.30	.764	-2.29	1.69
Age [= 8]	0.03	0.89	50.00	0.03	.970	-1.76	1.83
Age [= 9]	-0.24	0.84	50.00	-0.29	.771	-1.94	1.44
Age [= 10]	-1.81	0.89	50.00	-2.02	.048	-3.61	-0.01
Group [= 1]	2.01	1.59	50.00	1.26	.213	-1.19	5.22
Time [= t1] x Group [= 1]	-0.81	0.19	59.00	-4.28	.000	-1.23	-0.44
Age [= 6] x group [= 1]	-3.21	2.27	50.00	-1.41	.164	-7.79	1.35
Age [= 7] x group [= 1]	-2.31	1.88	50.00	-1.23	.224	-6.09	1.46
Age [= 8] x group [= 1]	-1.10	1.78	50.00	-0.61	.541	-4.69	2.48
Age [= 9] x group [= 1]	-1.07	1.73	50.00	-0.61	.540	-4.55	2.41

Random effect: intercepts and slopes for the time effect.

**Table 6:** Result of the linear mixed model for use of prepositions.

						*95% Confidence Interval*	
Parameter (fixed effects)	*β*	SE(*β*)	*df*	*T*	p	Lower	Upper
Intercept	2.93	0.69	56.03	4.21	.000	1.53	4.32
Time [= t1]	-0.35	0.21	59.00	-1.67	.100	-0.77	0.06
Age [= 6]	-0.75	1.64	50.00	-0.46	.646	-4.06	2.54
Age [= 7]	-0.15	1.00	50.00	-0.15	.875	-2.18	1.86
Age [= 8]	-1.52	0.90	50.00	-1.67	.100	-3.35	0.30
Age [= 9]	-0.78	0.85	50.00	-0.91	.364	-2.50	0.93
Age [= 10]	-1.13	0.90	50.00	-1.25	.216	-2.96	0.68
Group [= 1]	-0.87	1.64	52.36	-0.53	.595	-4.17	2.41
Time [= t1] x Group [= 1]	-0.55	0.29	59.00	-1.85	.068	-1.14	0.04
Age [= 6] x group [= 1]	-0.28	2.31	50.00	-0.12	.902	-4.92	4.35
Age [= 7] x group [= 1]	-0.05	1.90	50.00	-0.03	.976	-3.89	3.77
Age [= 8] x group [= 1]	1.85	1.81	50.00	1.02	.312	-1.79	5.49
Age [= 9] x group [= 1]	0.79	1.76	50.00	0.45	.654	-2.74	4.33

Random effect: intercepts and slopes for the time effect.

#### Domain specific vocabulary

**Fig 2:**
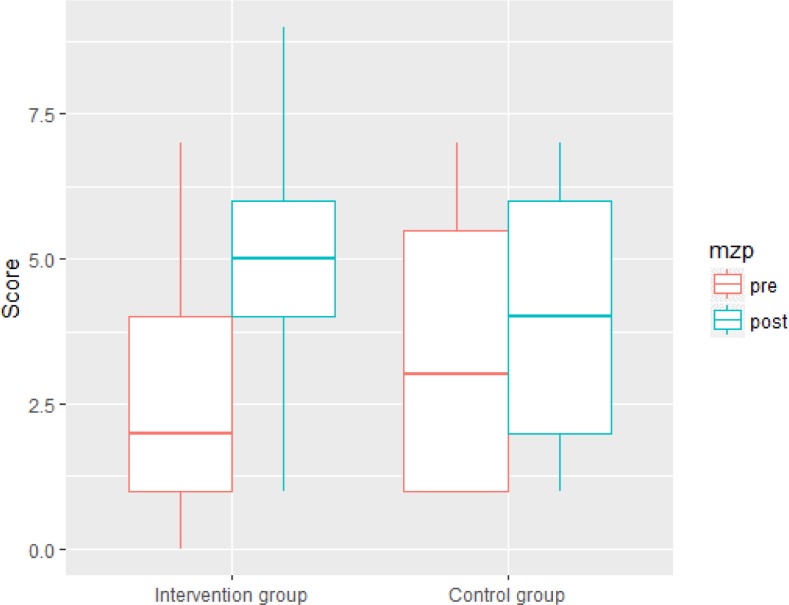
Group x time interaction effect on domain specific vocabulary.

The linear mixed effect model (Table 4) revealed a main effect of time but not of age and group. Domain specific vocabulary scores in posttests increased significantly in both groups but this increase was not affected by age nor group. We found the predicted two-way interaction between time and group. The increase in test scores on domain specific vocabulary was pronounced in the intervention group compared to the control group. The interaction between age and group was not significant. The significant interaction of time of measurement and group is shown in Fig 2.

#### Listening comprehension

**Fig 3:**
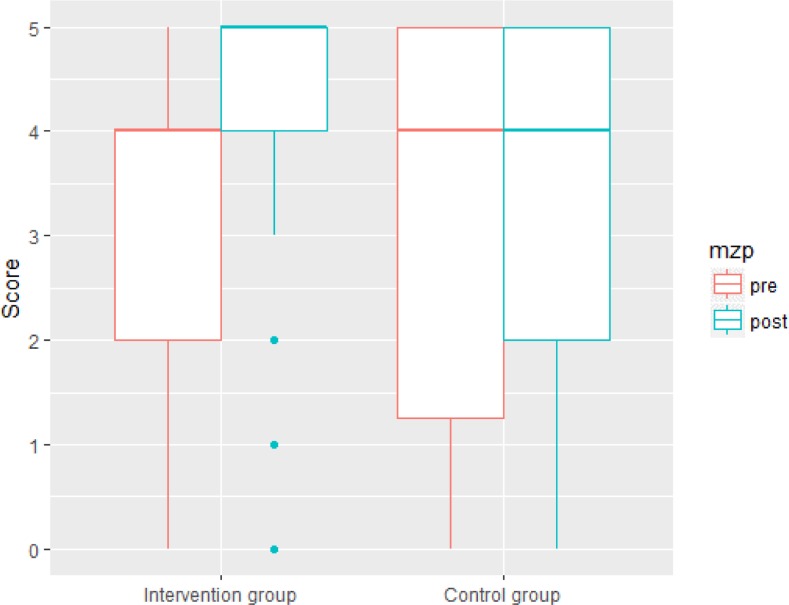
Group x time interaction effect on listening comprehension.

The linear mixed effect model (Table 5) revealed no main effects of time, age and group. Listening comprehension scores after the intervention were not affected by group and by age. Crucially, we found the predicted two-way interaction between time and group. The increase in listening comprehension scores of the intervention group was more pronounced compared to the increase in the control group. The interaction between age and group was not significant. The significant interaction of time and group is shown in Fig 3.

#### Use of local prepositions

The linear mixed effect model (Table 6) revealed no main effect of time, age and group. The use of local prepositions following the intervention period was not affected by group or by age. We did not find the predicted two-way interaction between time of measurement and group. Also, the interaction between age and group was not significant.

## Discussion and conclusions

The aim of the present pilot study was to analyze second language acquisition effects of an intPE-L2 intervention for young refugees using a quasi-experimental design that referred to an established theoretical framework and included a control group. Our hypothesis postulated that the intervention would be associated with higher improvements regarding domain specific vocabulary learning, listening comprehension, and use of local prepositions within the context of primary PE games focusing on tossing and running rather than schooling and maturation only. The hypothesis was partly confirmed in accuracy measures in the domain specific vocabulary learning and listening comprehension tests where the intervention group showed better performance compared to the control group. No significant effect was found for the use of local prepositions. This suggests that the intPE-L2-intervention improved domain specific vocabulary and listening comprehension within the context of primary PE games for the target group beyond maturation and schooling. These findings are in line with earlier findings obtained from other L2-learners from several pre-school samples [9–11]. The missing effect on use of local prepositions beyond maturation and schooling may be due to the circumstance that teaching the use of local prepositions was not intense or domain specific enough to consistently affect this specific language skill. Furthermore, training the use of local prepositions is normally integrated in additional German language learning activities for this target group in extracurricular L2-learning lessons at this time of their school career. Therefore, we plan to explore in more detail to what extent the intPE-L2-intervention can improve the use of local and other prepositions beyond schooling and maturation in further studies. For this, the integration of more difficult test items might be beneficial to differentiate between groups. Also, video coding of intervention lessons could be used to examine the right amount of teaching intensity by analyzing the frequency of relevant elements (e.g. words, sentences). In addition, in future intPE-L2-interventions that will exceed the character of a pilot study we aim to test the children with a larger number of test items in domain specific vocabulary, listening comprehension and use of prepositions.

Albeit preliminary, the results of the present study suggest specific effects of intPE-L2-intervention on young refugees at primary school level on domain specific vocabulary learning and listening comprehension. Nevertheless, the findings should be treated with care, because they are grounded on a relatively small sample. Furthermore, the evidence is limited by the fact that the participants were not randomly assigned to the study groups. However, the pattern of results encourages further studies with larger randomized and controlled trials to test the replicability of effects and to test long-term effects. Also, it might be interesting to look at transfer effects in the future, i.e. whether the L2 learning that takes place during PE classes also affects the children’s performance during other school lessons. Beside successful replication, the mechanisms underlying these effects deserve further empirical investigation.

A validation of our results with refugees from different schools and other age groups might also be desirable. Future intPE-L2-intervention studies based on the same and other PE content might strengthen the evidence. It should also be noted that the present study was conducted with young refugees from diverse national, cultural, and religious backgrounds. If the results are successfully replicated in future studies and in view of the existing evidence concerning positive effects of intPE-L2-intervention, there is good reason to establish this approach as one effective domain specific L2-learning strategy within the PE context with the target group of refugees. Further studies should check whether these effects could also be transferred to intPE-L1-learning scenarios when participants do not have a L2-learning background.

## Supporting information

S1 File ag_intervention_arno.sav. (SAV)
